# Exploring the Advantages of a Hydrolyzed Rice Formula in the Dietary Management of Infants with Cow’s Milk Allergy in the Middle East, North Africa, and Pakistan Region

**DOI:** 10.3390/nu13103429

**Published:** 2021-09-28

**Authors:** Yvan Vandenplas, Christophe Dupont, Wajeeh Al-Dekhail, Hani A. Al Hashmi, Ahmed Fouad Khalil, Mostafa Abdel-Aziz El-Hodhod, Khaled Husain, Avantika Singh

**Affiliations:** 1 Vrije Unversiteit Brussel (VUB), UZ Brussel, KidZ Health Castle, 1090 Brussels, Belgium; 2 Pediatric Gastroenterology Department, Paris-Descartes University, 75006 Paris, France; christophe.dupont@wanadoo.fr; 3 Pediatric Gastroenterology Department, APHP Necker-Enfants Malades Hospital, 750015 Paris, France; 4 Marcel Sembat Clinic, 92100 Boulogne, France; 5 Section of Gastroenterology, Pediatrics Department, King Faisal Specialist Hospital & Research Centre, P.O. Box 3354, Riyadh 11211, Saudi Arabia; wdekhail@kfshrc.edu.sa; 6 Section of Allergy and Clinical Immunology, Pediatrics Department, King Abdulaziz Hospital, Jeddah 22421, Saudi Arabia; aboabdullahha777@gmail.com; 7 Pediatric Gastroenterology and Nutrition Department, Alexandria University, Alexandria 21544, Egypt; fouad_ped@yahoo.com; 8 Pediatric Gastroenterology and Endoscopy Department, Faculty of Medicine, Ain Shams University, Cairo 11535, Egypt; mostafaelhodhod@yahoo.com; 9 Dean of Faculty of Medicine, October 6 University, Giza 12511, Egypt; 10 Pediatric Gastroenterology, Hepatology and Nutrition Department, Dar AlShifa Hospital, Kuwait City 13034, Kuwait; khusain12@yahoo.com; 11 Pediatric Gastroenterology Department, Mediclinic Airport Road, Abu Dhabi 48481, United Arab Emirates; Avantika.Singh@mediclinic.ae

**Keywords:** cow’s milk allergy, hydrolyzed protein, infant allergy, infant feeding, Middle East, rice

## Abstract

Cow’s milk allergy (CMA) is the most common food allergy in early childhood, and its prevalence continues to rise. Exclusive breastfeeding is recommended for infants in the first 6 months of life, but this recommendation is poorly adhered to in many parts of the world, including the Middle East, North Africa, and Pakistan (MENAP) region. If the infant is affected by CMA, current guidelines recommend extensively hydrolyzed formulas (eHFs) or amino acid-based formulas (AAFs) in the case of severe symptoms, and hydrolyzed rice formulas (HRFs) where available. In recent years, HRFs have been proposed as a plant-based alternative to cow’s milk protein-based eHFs, and updates to current guidelines have been recommended. In 2014, a consensus statement and guidelines were published for the Middle East region on the prevention, diagnosis, and management of CMA. As new advances have been made in the extensively hydrolyzed hypoallergenic infant formula space, along with updated scientific evidence, a workshop of experts from the MENAP region focused on HRF was convened in 2021. This publication summarizes the insights from this meeting. During the consensus part of the meeting, a new approach was discussed and approved by all participants, and agreement was reached that HRF can be recommended as a first-line alternative to cow’s milk-based eHF in the dietary management of CMA.

## 1. Introduction

Allergy risk has become a significant public health issue with rising prevalence, and despite past, current, and ongoing research, the reasons are not fully understood [1,2,3]. In infants, exclusive breastfeeding is recommended for the first 6 months of life, but this recommendation is poorly adhered to in many parts of the world, including the Middle East, North Africa, and Pakistan (MENAP) region, putting infants at a possible risk of developing allergic sensitization and disorders [3]. The MENAP region remains very heterogeneous with different levels of clinical practice as allergic disorders may be seen by healthcare professionals (HCPs) spanning multiple specialties, such as general pediatrics, pulmonology, dermatology, gastroenterology, and allergy/immunology. A wide range of dietary management approaches exists within the region itself, including the use of partially hydrolyzed whey formulas (pHF-Ws) [3].

Cow’s milk allergy (CMA) is the most common food allergy in early childhood, and approximately 80% of children will outgrow it by 3–5 years of age [4,5]; its prevalence ranges from 1.9% to 4.9% [6]. It manifests through a variety of symptoms that place a significant burden on the infant and caregiver alike [7]. If the infant is affected by CMA, expert guidelines recommend cow’s milk-based extensively hydrolyzed formulas (eHFs) or amino acid-based formulas (AAFs) in the presence of severe symptoms [3]. In 2010, the World Allergy Organization (WAO) published the Diagnosis and Rationale for Action against Cow’s Milk Allergy (DRACMA) guidelines [8]. In recent years, hydrolyzed rice formulas (HRFs) have been proposed as a suitable plant-based alternative to cow’s milk protein-based eHFs, and updates to current guidelines have been recommended by DRACMA and the Committee on Nutrition of the French Society of Pediatrics [9,10]. Several studies have shown the feasibility of HRF in treating CMA, ensuring satisfactory growth from the first year of life for infants and toddlers [9]. In 2014, a consensus statement and guidelines were published, setting out best practice for the Middle East region on the prevention, diagnosis, and treatment of CMA [7]. In 2019, the Middle-East Step-Down approach described the management of CMA using pHF-W as a bridge between eHF or AAF and intact CMP to make clear that pHF-W has no indication in the management of CMA [3].

As new advances have been made in extensively hydrolyzed hypoallergenic infant formulae, along with updated scientific evidence, a workshop of experts from the MENAP region focused on HRF was convened in 2021. This publication summarizes the presentations, discussions, and findings from this meeting, discussing the advantages of HRF in the dietary management of infants with CMA. During the consensus section of the meeting, a potential new approach to the therapeutic management of CMA using HRF was discussed and approved by all participants.

## 2. Background: MENAP Workshop

On Friday, 9 April 2021, a virtual roundtable was held for HCPs from the MENAP region (five pediatric gastroenterologists and one pediatric allergist/immunologist). Representative countries included Egypt, Kuwait, Saudi Arabia, and the United Arab Emirates. The meeting was chaired by Yvan Vandenplas, Head of the Department of Paediatrics, “KidZ Health Castle”, at the University Hospital Brussels in Belgium. The primary goal was to align on challenges with currently prescribed first-line formulas for CMA dietary management and as part of a diagnostic elimination diet (Box 1).

Box 1 **Meeting objectives/goals.**

1. Align on challenges with formulas currently prescribed first line for CMA dietary management and as part of a diagnostic elimination diet, leveraging findings from a recent MENAP HCP survey.
2. Define the evidence-based benefits of HRF compared with cow’s milk protein-based eHF.
3. Highlight the drawbacks and limitations of pHF-W and why its use should be limited in the prevention of CMA.
4. Align on a set of recommendations HCPs can use to determine when and where to initiate HRF.

Legend: CMA, cow’s milk allergy; eHF, extensively hydrolyzed formula; HCP, healthcare professional; HRF, hydrolyzed rice formula; pHF-W, partially hydrolyzed whey formula.

This roundtable built on a previous event hosted in August 2020 with some of the same participants, which focused on introducing HRF in general and raising awareness for its evidence-based benefits as a plant-based alternative to cow’s milk protein-based eHF.

## 3. Summary of 2021 Workshop

The workshop was divided into three sections: the presentation and discussion of HCP survey findings from the MENAP region, an expert presentation from Christophe Dupont (on his clinical experience with HRF from a gastroenterologist’s perspective), and a facilitated discussion on consensus statements on the use of HRF by HCPs in the MENAP region.

### 3.1. HCP Survey Findings from the MENAP Region

A brief survey of six questions was distributed to 50 HCPs (general pediatricians) with an interest in infant nutrition and cow’s milk allergy in Kuwait, Saudi Arabia, and the United Arab Emirates in advance of the roundtable, to assess current first- and second-line approaches to the dietary management of CMA. Responses (n = 43/50; 86%) were collected between 11 February and 25 March 2021. Key findings are presented in Table 1.

Access to and the availability of HRF remains the biggest barrier to its widespread usage by physicians in the MENAP region. Additionally, the fact that one third of HCPs surveyed are prescribing pHF-W to their patients despite current guideline recommendations may illustrate that a lack of understanding exists on the difference in hydrolysis between pHF-W and eHF, and also that HRF category-specific education is needed in this region, starting at the first-line prescriber level. The MENAP expert attendees described affordability, palatability, and clinical response as the most important criteria to them when considering switching to a different formula.

### 3.2. Expert Presentation: Practical Experience Using HRF from a Gastroenterologist’s Perspective

Christophe Dupont presented his clinical experience with HRF from a gastroenterologist’s perspective. He briefly overviewed HRF, the rationale for its first- and second-line use, and introduced three clinical case studies. The attendees discussed whether HRF could be used first line and debated the inclusion of HRF in an elimination diet as part of the CMA diagnostic work-up. They discussed the lack of market availability of HRF in the MENAP region. It is not a quick process to change HCP prescribing behavior, as evidenced by the length of time it has taken for HRF to become mainstream practice in some European countries. Indeed, HRF is currently marketed mainly in Italy, France, and Spain [6].

### 3.3. Consensus Recommendations

For the development of a regional consensus on the use of HRF for CMA in the MENAP region, a structured quantitative method was used to facilitate the discussion and reach a consensus [11]. Statements were prepared before the meeting by the lead author, based on his clinical experience acquired from previous guidelines he co-authored for the MENAP region [3,7]. At the meeting, each statement was discussed comprehensively within the group. All group members (n = 6) then voted anonymously on each statement (using an online voting Zoom poll by clicking a box for “agreement” or “disagreement.” Poll results were tallied). For any disagreements, the statements were revised accordingly, and a second vote was taken.

Ultimately, there was strong belief among MENAP experts in the evidence-based benefits of HRF based on full consensus on nearly all statements presented (Table 2). An exception to immediate full consensus was one minor edit to statement 11 regarding the percentage of infants with IgE- and non-IgE-mediated CMA who may react to eHF (the percentage of infants with non-IgE-mediated CMA was changed from “30%” to “15% up to 30%”).

Local clinical data/experience will be essential to increase physician confidence in regions where HRF is not currently an option (e.g., Kuwait, Saudi Arabia, United Arab Emirates), as will endorsement through potential third parties, as that level of credibility resonates with this audience. It will be critical to raise awareness and disseminate knowledge among a variety of HCPs via scientific meetings, presentations, and community webinars, and to concisely communicate the evidence-based benefits of HRF in an easy to digest format (e.g., infographics, digital, social media). Local-level efforts will be important education avenues once COVID-19 restrictions ease to increase confidence and help change recommending behavior in the MENAP region among all levels of HCPs—from first-line prescribers to specialist pediatricians.

## 4. Conclusions

A summary of key findings from this workshop is reported below (Box 2). This roundtable meeting illustrated that HRF has the potential to be widely used and accepted in the MENAP region in place of eHF due to improved palatability and other compelling attributes, such as competitive cost and being free from residual cow’s milk protein. Given that the previous Middle East consensus statements do not mention HRF [7], the consensus statements developed and ratified in this roundtable are a crucial first step in helping to drive clinical practice change once HRF is readily available. Education efforts, ideally led by third-party experts, are a key factor to expediting the widespread use and acceptance of HRF in the MENAP region. The consensus statements developed at this meeting are based on expert opinion and advocate the place of HRF in the dietary management of CMA.

Box 2 **Summary of findings from a workshop exploring the advantages of an HRF in the dietary management of infants with CMA in the MENAP region.**

1. Many HCPs surveyed in the MENAP region prescribe pHF-W to their patients despite local guideline recommendations, illustrating that HCPs may not be properly confirming a CMA diagnosis before making a formula recommendation.
2. Compared with eHF, HRF has better palatability and is free from residual cow’s milk protein.
3. Access and availability are still seen as the biggest barriers to widespread usage in the region.
4. For regions where HRF is not currently available as a treatment option for CMA (e.g., Kuwait, Saudi Arabia, United Arab Emirates), local clinical data, real-world case studies, and practical experience will be essential to increase physician confidence in recommending HRF.
5. Third-party credibility resonates with this audience, as the questions asked of Dr. Vandenplas and Dr. Dupont illustrated a desire to learn more about HRF and their clinical experience.
6. Given that the previous Middle East consensus statements do not mention HRF (Vandenplas 2014), these new consensus statements will be a crucial first step in helping to drive clinical practice change once HRF is readily available.
7. There was consensus on nearly every single statement presented (except a minor edit to statement 11). This shows that there is a strong belief in the evidence-based benefits of HRF and that once availability and education intersect, there is tremendous potential for HRF to be used first line as a CMA dietary management option.
8. Once HRF becomes more readily available in the MENAP region and first-line prescribers become more familiar with its benefits, there is potential for HRF uptake as a first-line CMA dietary management option.

Legend: eHF, extensively hydrolyzed formula; CMA, cow’s milk allergy; GI, gastrointestinal; HCP, healthcare professional; HRF, hydrolyzed rice formula; MENAP, Middle East, North Africa, and Pakistan; pHF-W, partially hydrolyzed whey formula.

## Figures and Tables

**Table 1:** Top-line HCP survey findings of current approaches to dietary management of CMA from the MENAP region.

**First Line**
eHF (53%) is selected first line by HCPs to manage mild-to-moderate IgE- and non-IgE-mediated CMA. Poor palatability and formula acceptance were the most frequent challenges faced by patients utilizing AAF and eHF. Lack of efficacy was the most frequent challenge faced by patients utilizing pHF-W to feed children with confirmed CMA. Nearly all (97%) respondents indicated that patients at least sometimes switch from a first-line formula; 68% of HCPs indicated that patients “sometimes” make the switch, 24% indicated this happens “often”, and 5% indicated “very often”.
**Second Line**
49% of HCPs initiated AAF and 41% of HCPs initiated pHF-W second line to manage mild-to-moderate CMA. HCPs indicated challenges with formula acceptance in patients utilizing eHF and issues with affordability/cost. 69% of HCPs indicated that patients “sometimes” switch from a second-line formula, 14% “often” make this switch, while 14% “never” switch.

AAF, amino acid formula; CMA, cow’s milk allergy; eHF, extensively hydrolyzed casein/whey formula; HCP, healthcare professional; Ig, immunoglobulin; pHF-W, partially hydrolyzed whey formula.

**Table 2:** MENAP consensus statements on the use of HRF.

No.	Statement	Agreement
1	For formula-fed infants, the principle of the management of CMA is to provide infants with a feed tolerated by the immune system, thus preventing the immune system from being in contact with cow’s milk peptides, which are not tolerated.	100%
2	An elimination diet lasting 2–4 weeks with an extensively hydrolyzed cow’s milk-based formula is now recommended in the guidelines if mild-to-moderate CMA is suspected in formula-fed infants <12 months old.	100%
3	A challenge test (reintroduction of cow’s milk protein) is the scientific recommendation to confirm the diagnosis of mild-to-moderate CMA.	100%
4	A challenge test (reintroduction of cow’s milk protein) is recommended to confirm the diagnosis of mild-to-moderate CMA, but is often refused by parents in clinical practice.	100%
5	An elimination diet lasting 2–4 weeks with an AAF is recommended if severe (faltering growth, anaphylaxis) CMAis suspected in formula-fed infants <12 months old.	100%
6	Partially hydrolyzed cow’s milk-based formulas are not indicated in the management of CMA *.	100%
7	HRFs have been available for over 20 years and have been shown to be nutritionally adequate and safe.	100%
8	HRFs have been shown to be effective in the management of mild-to-moderate CMA.	100%
9	Due to their limited geographical availability, HRFs have historically not been considered in guidelines.	100%
10	Based on the available nutritional studies and efficacy studies in CMA, as well as their increased geographical availability, HRFs can be recommended for the management of mild-to-moderate CMA.	100%
11	HRFs might be more effective than cow’s milk-based eHFs, since on average 12% of infants with IgE-mediated CMA and 15% up to 30% of infants with non-IgE-mediated CMA may react to eHFs.	100%
12	HRFs are considered to be more palatable than eHFs.	100%
13	Per definition, HRFs are cow’s milk protein-free.	100%
14	An elimination diet lasting 2–4 weeks with HRF is recommended if mild-to-moderate CMA is suspected in formula-fed infants <12 months old.	100%
15	HRF can be recommended as a first-line option.	100%
16	HRFs are suitable for those following a Halal diet ^†^.	100%

AAF, amino acid formula; CMA, cow’s milk allergy; eHF, extensively hydrolyzed formula; HRF, hydrolyzed rice formula; Ig, immunoglobulin; pHF-W, partially hydrolyzed whey formula. * The Middle East region has a unique practice of utilizing pHF-W as a step-down between eHF or AAF and intact CMP. However, strict protocols need to be adhered to, including a carefully conducted pHF-W challenge test, before initiating this approach [3]. ^†^ Please check with the infant formula manufacturer to ensure a product is certified Halal.

## Data Availability

Not applicable.
